# Knowledge Mapping of Drug-Induced Liver Injury: A Scientometric Investigation (2010–2019)

**DOI:** 10.3389/fphar.2020.00842

**Published:** 2020-06-05

**Authors:** Lixin Ke, Cuncun Lu, Rui Shen, Tingting Lu, Bin Ma, Yunpeng Hua

**Affiliations:** ^1^Department of Liver Surgery, The First Affiliated Hospital, Sun Yat-sen University, Guangzhou, China; ^2^Evidence-Based Medicine Center, School of Basic Medical Sciences, Lanzhou University, Lanzhou, China; ^3^Key Laboratory of Preclinical Study for New Drug of Gansu Province, School of Basic Medical Science, Lanzhou University, Lanzhou, China

**Keywords:** scientometric, drug-induced liver injury, VOSviewer, CiteSpace, HLA-B*5701

## Abstract

**Background:**

Drug-induced liver injury (DILI) is a common adverse event, which compromises the safety of numerous drugs, poses a significant risk to patient health, and enhances healthcare expenditures. Many articles have been recently published on DILI related research, though no relevant scientometric study has been published yet. This scientometric study was aimed at comprehensively analyzing the knowledge base and emerging topics on DILI.

**Methods:**

The articles and reviews related to DILI, published from 2010 to 2019 in the Web of Science Core Collection (WoSCC), were retrieved on March 15, 2020, using relevant keywords. Four different scientometric software (HistCite, VOSviewer, CiteSpace, and R-bibliometrix) was used to conduct this scientometric study.

**Results:**

A total of 1,995 publications were retrieved (including 1,550 articles and 445 reviews) from 592 academic journals with 56,273 co-cited references in 10 languages by 2,331 institutions from 79 countries/regions. The majority of publications (*n* = 727, 36.44%) were published in the United States, and the University of North Carolina contributed the most publications (*n* = 89, 4.46%). The most productive academic journal on DILI was the *Toxicological Sciences* [*n* = 79, 3.96%; impact factor (IF) 2018 = 3.564], and *Hepatology* was the first co-cited journal (*n* = 7,383, IF 2018 = 14.971). Fontana RJ and Teschke R may have significant influence on DILI research, with more publications (*n* = 46; *n* = 39) and co-citations (*n* = 382; *n* = 945). Definition, incidence rate or clinical characteristics, etiology or pathogenesis (such as the character of the innate immune system, the regulation of cell-death pathways, and susceptible HLA-B*5701 genotype), identification of main drugs and causality assessment (criteria and methods) were the knowledge base for DILI research. Exploring the microscopic mechanism (such as the organelle dysfunction and cytotoxicity induced by drugs, and exploration of role of neutrophils in DILI using mouse models) and developed newer approaches to prevent DILI (such as the prospective HLA-B*5701 screening and *in vitro* approaches for assessing the potential risk of candidate drugs for DILI) were the recent major topics for DILI research.

**Conclusion:**

This scientometric study comprehensively reviewed the publications related to DILI during the past decade using quantitative and qualitative methods. This information would provide references for scholars, researching on DILI.

## Introduction

Drug-induced liver injury (DILI) is a common adverse event, which compromises the safety of numerous drugs and herbs, poses a significant risk to patient health, and enhances healthcare expenditures. Recently, DILI has received sustained attention from the patients, clinicians, and medical companies. Approximately, 139.0–240.0 per million of the global population suffer every year from drug-induced hepatotoxicity (including drugs, toxins, and herbal medicines) (Suk and Kim, 2012). In China, hepatotoxicity (induced by drugs, herbs, and toxins) comprises the majority of the cases of acute hepatic failure (Zhao et al., 2013). Annually, DILI occurs to every 238.0 patients per million of the Chinese population (Shen et al., 2019). In Western countries, DILI comprises most of the acute hepatic failure cases (Ostapowicz et al., 2002; Wei et al., 2007; Reuben et al., 2010). The total number of global registries and series was approximately 12 as some updated case series of DILI arose in 2015 (Lewis, 2015; Andrade et al., 2016). Chalasani et al. collated all the information related to the cases of DILI during the last 10 years from the United States DILI Network (DILIN) (Chalasani et al., 2015). They analyzed the first 1257 patients and marked 899 of these patients as possible DILI cases. DILI is the major reason behind the rejection of an investigational drug, leading to the failure of a clinical trial (Kullak-Ublick et al., 2017). From 1969 to 2002, 15% of drugs were withdrawn from the market due to hepatotoxicity (Wysowski and Swartz, 2005). In the future, more cases of DILI may be reported as the use of drugs and herbs may increase, especially among the elderly population. The diagnosis and management of DILI are extremely challenging tasks due to 1) the unavailability of precise diagnostic markers for excluding other causes of hepatic injury (Kullak-Ublick et al., 2017) and 2) the availability of countless hepatotoxic drugs (Hoofnagle and Björnsson, 2019).

Scientometric analysis can quantify the impact of individual research results and the literature development of specific subjects and evaluate the tendencies of scientific research (Chen et al., 2015; Gao et al., 2019; Lu et al., 2019a; Garas et al., 2020). It focuses on the metrological features of literature (Ellegaard and Wallin, 2015) and identifies different characteristics, like countries, institutions, journals, authors, and keywords of various publications in a particular field over a period (Gao et al., 2019), so that the researchers can summarize the current situation and development trends of a research field or a specific disease and provide directions and ideas for future research (Lu et al., 2019a). Generally, the scientometric analysis consists of three steps: 1) obtaining the publications from accessible databases, 2) conducting analysis by software tools, and 3) writing the manuscript for publication. The Web of Science Core Collection (WoSCC) is the most preferred database for scientometric analyses (Miao et al., 2018; Gao et al., 2019; Huang et al., 2019), and the current software for scientometric analysis includes HistCite (Garfield et al., 2006), VOSviewer (van Eck and Waltman, 2010), CiteSpace (Chen, 2006), and R-bibliometrix (Aria and Cuccurullo, 2017). The scientometric analysis also identifies development trends and research hotspots in many fields (Garas et al., 2020; Miao et al., 2018; Gao et al., 2019; Lu et al., 2019a). Using this method, Lu *et al*. (Lu et al., 2020) reported that currently the researches on *Angelica sinensis* (AS) were focused on identifying and assessing its active components (like ferulic acid) and pharmacological actions (such as immunomodulatory effects). Miao et al. (Miao et al., 2018) analyzed the researches on hepatocellular carcinoma, especially those primarily concentrated on the transarterial chemoembolization, epithelial-mesenchymal transition and cancer stem cell. However, no specific scientometric investigation has yet been conducted on the knowledge mapping of DILI. Thus, our study was aimed at comprehensively analyzing the knowledge base and emerging information on DILI.

## Materials and Methods

### Data Source and Search Strategy

We searched WoSCC on March 15, 2020, at Sun Yat-Sen University to collate recent DILI-related studies between 2010 and 2019. The database source was limited to Science Citation Index Expanded (SCIE) and publication types to “article” or “review”. The main search terms were as described below: “drug induced liver injury”, “drug induced liver damage”, “drug induced liver diseases”, “drug induced liver hepatitis”, “drug induced liver cirrhosis”, and “drug induced liver failure”, etc. The detailed search strategy has been attached to **Supplementary Material**. For the accuracy of data, we downloaded all eligible data from the WoSCC on March 15, 2020, and further analyzed by scientometric tools.

### Statistical Analysis

HistCite (12.03.07) (Garfield et al., 2006) was used to identify the annual output (number of publications), publication languages, and publication types. The IFs of the academic journals were collected from the 2018 Journal Citation Reports (JCR) (Clarivate Analytics, Philadelphia, USA). VOSviewer (1.6.13) was used to identify productive countries/regions, institutions, journals and authors, as well as main co-cited journals, authors and references, and related visual networks were also constructed (van Eck and Waltman, 2010; Gao et al., 2019). Also, we constructed the network of regions distribution for DILI-related publications using R–bibliometrix (Aria and Cuccurullo, 2017). In the VOSviewer network maps, different nodes indicate components, such as countries/regions, institutions, and journals. The size of the nodes reflects the number of studies or cooccurrence frequencies. The links between nodes represent the cooccurrence relationships, and the size of the links indicates the cooccurrence frequencies of two nodes (Lu et al., 2019a). The VOSviewer settings were as follows: counting method (full counting), while, thresholds (T) of items (countries/regions, institutions, journals, authors, and references) were adopted based on special situations. CiteSpace (5.6.R2) (Chen, 2006) explores the tendencies and dynamics of scientific studies in a given research field (Chen, 2004; Li et al., 2017), and we used it to construct the dual-map overlay for journals and detected the references with strong citation burstness to identify the emerging topics. The CiteSpace parameters were as follows: link retaining factor (LRF = 3), look back years (LBY = −1), e for top N (e = 2), time span (2010–2019), years per slice (1), links (strength: cosine, scope: within slices), selection criteria (g-index: k = 25), and minimum duration (MD = 5). We managed the data and analyzed the publication trend using Microsoft Office Excel 2019 (Microsoft Corporation, Redmond, WA, United States). The linear model f (x) = ax+b was used to predict the number of studies in 2020. Variable f (x) represents the number of studies, and x denotes the publication year.

## Results

### Annual Growth Trend of Publications

**Figure 1:**
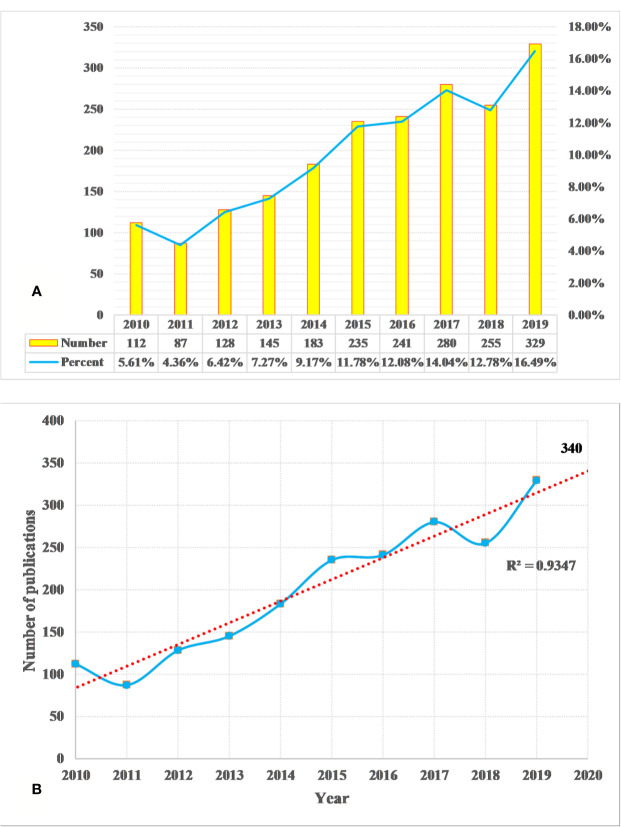
Annual output of drug-induced liver injury (DILI) research **(A)** and the linear fitting of publication growth in DILI research **(B)**.

We identified a total of 1,995 DILI studies, published between 2010 and 2019, including 1,550 articles and 445 reviews. A total of 10 languages were used in the identified publications, including English (*n* = 1,964, 98.45%), German (*n* = 12, 0.60%), Spanish (*n* = 6, 0.30%), Japanese (*n* = 4, 0.20%), Chinese (*n* = 2, 0.10%), French (*n* = 2, 0.10%), Russian (*n* = 2, 0.10%), Icelandic (*n* = 1, 0.05%), Portuguese (*n* = 1, 0.05%), and Slovenian (*n* = 1, 0.05%). In **Figure 1A**, the annual output of DILI-related studies shows an upward trend during the period from 2010 to 2019. The annual output was more than 100 papers in the past years, but the least number of papers were published in 2011 (*n* = 87, 4.36%), and the average annual output was about 200. Over 200 papers have been published annually since 2015 (*n* = 235, 11.78%), which reached the maximum in 2019 (*n* = 329, 16.49%). The linear fitting of DILI-related studies shows a significant correlation (the coefficient of determination (R^2^) = 0.9347) between the publication year and the number of studies (**Figure 1B**). According to linear fitting, the number of studies will reach about 340 in 2020.

### Countries/Regions and Institutions Analysis

**Figure 2:**
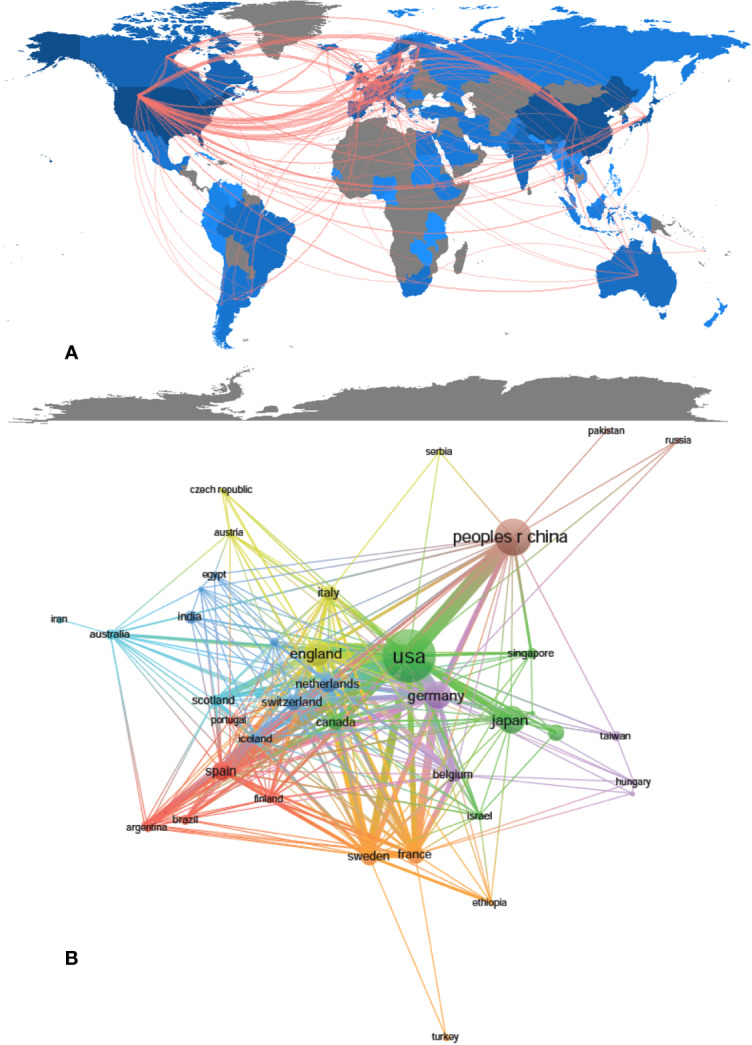
The regional distribution **(A)** and the network map of countries (**B**, T = 5) related to drug-induced liver injury (DILI) research.

A total of 1,995 publications were coauthored by 2,331 institutions from 79 countries/regions. The top 10 countries/regions and institutions are shown in **Table 1** according to the publication number. The top 10 countries/regions are distributed in three continents, half of which are distributed in Europe (**Figure 2A**). The publications from five countries/regions were less than 100: Spain (*n* = 97); France (*n* = 79); Netherlands (*n* = 77); Sweden (*n* = 69) and Canada (*n* = 66). The rest were more than 175 (England), and the largest number of papers were originated from the United States (*n* = 727), followed by China (*n* = 347), Japan (*n* = 187), and Germany (*n* = 186) (**Table 1**). According to **Figure 2B**, the countries/regions (45/79, 56.96%) with the publication number greater than or equal to 5 (T = 5) were used to construct the co-authorship network. In this network map, the United States, China, Japan, and Germany have larger sizes of nodes, representing more publications. Many active collaborations were noted among different countries/regions. For example, the United States has close cooperation with China, Germany, England, Australia, etc; Germany has cooperation with China, Switzerland, Sweden, France, etc.

**Table 1:** The top 10 countries/regions and institutions involved to DILI research.

Rank	Country/Region	Count	Institution	Count
1	The United States (North America)	727	University of North Carolina (United States)	89
2	China (Asia)	347	University of Michigan (United States)	64
3	Japan (Asia)	187	University of Liverpool (England)	60
4	Germany (Europe)	186	U.S. Food and Drug Administration (United States)	58
5	England (Europe)	175	Indiana University School of Medicine (United States)	46
6	Spain (Europe)	97	Duke University (United States)	45
7	France (Europe)	79	National Institute & Diabetes and Digestive and Kidney (United States)	39
8	Netherlands (Europe)	77	University of Malaga (Spain)	38
9	Sweden (Europe)	69	University of Toronto (Canada)	37
10	Canada (North America)	66	Kanazawa University (Japan)	33

**Figure 3:**
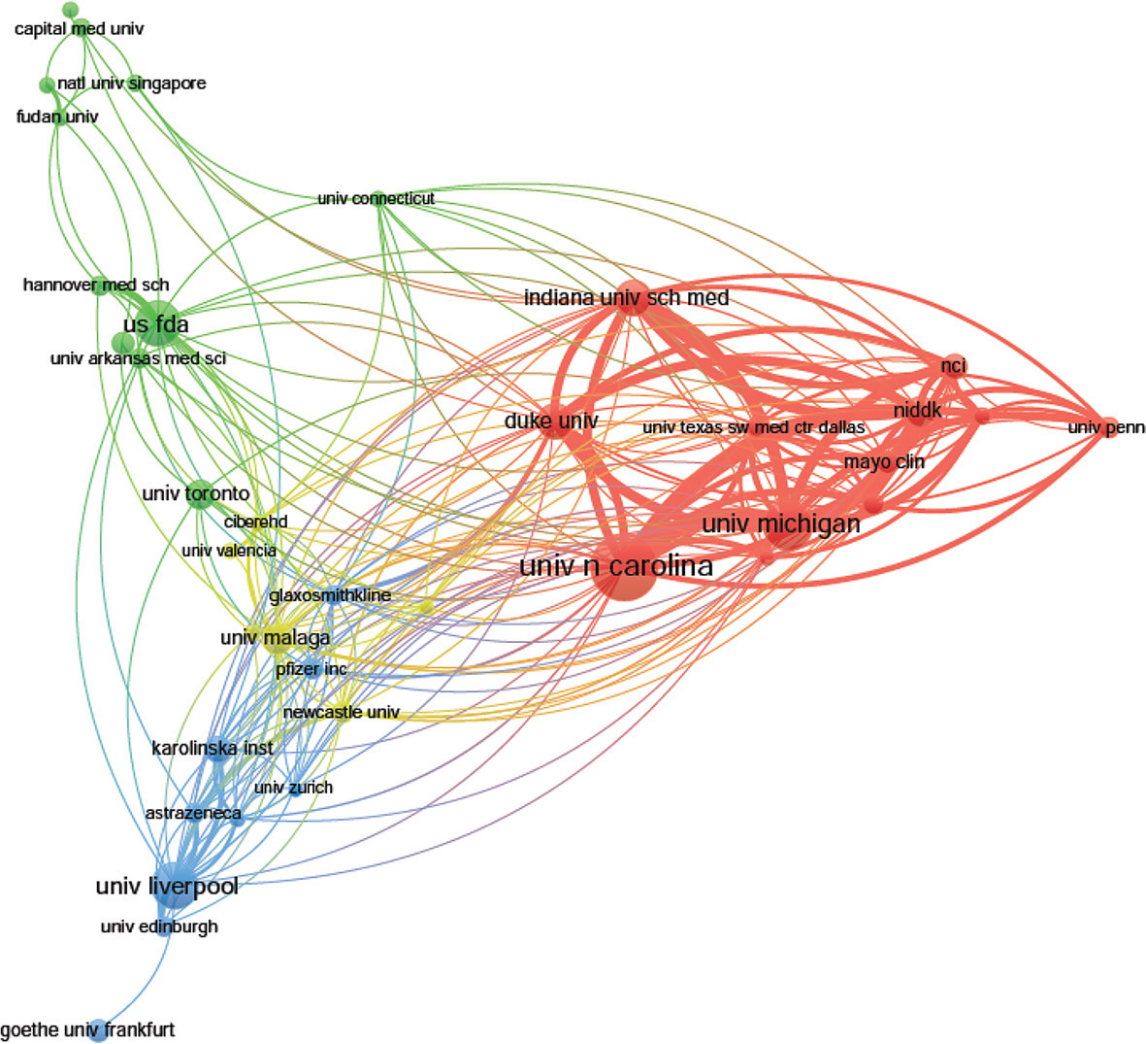
The network map of institutions for drug-induced liver injury (DILI) research (T = 17).

The top 10 institutions are distributed in five countries/regions, three-fifths of which are located in the United States. A total of four institutions published more than 50 papers: University of North Carolina (*n* = 89); University of Michigan (*n* = 64); University of Liverpool (*n* = 60), and U.S. Food and Drug Administration (*n* = 58). The rest are less than 46 (Indiana University School of Medicine): Duke University (*n* = 45), National Institute & Diabetes and Digestive and Kidney (*n* = 39), University of Malaga (*n* = 38), University of Toronto (*n* = 37), and Kanazawa University (*n* = 33) (**Table 1**). According to **Figure 3**, institutions (41/2,331, 1.76%) with more than or equal to 17 (T = 17) publications were used to construct the co-authorship network, and the largest subnetwork was presented. Within this visual network, the sizes of the nodes of the University of North Carolina, the University of Michigan, and the University of Liverpool are larger (because they published more studies). Many active collaborations were noted among different institutions. For example, the University of North Carolina has close cooperation with the University of Michigan, National Institute & Diabetes and Digestive and Kidney, Indiana University School of Medicine and Duke University, etc. The University of Liverpool has active cooperation with the University of Edinburgh, Karolinska Institute, and AstraZeneca, etc.

### Journals and Co-Cited Academic Journals

**Figure 4:**
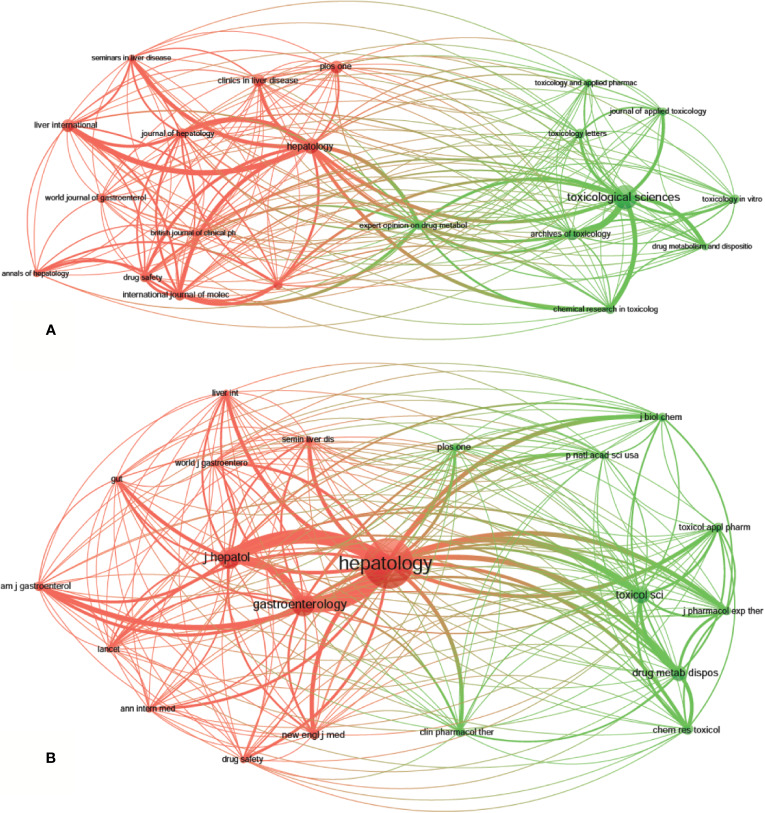
The network map of academic journals (**A**, T = 21) and co-cited academic journals (**B**, T=700) for drug-induced liver injury (DILI) research.

A total of 1,995 publications, related to DILI, were published in 592 academic journals. Over 35 papers were published in six journals, and all of them were in the United States, except for *Archives of Toxicology* in Germany (**Table 2**). *Toxicological Sciences* published the most papers (*n* = 79, IF2018 = 3.564, Q1), followed by *Hepatology* (*n* = 48, IF2018 = 14.971, Q1), *Liver International* (*n* = 38, IF2018 = 5.542, Q1), *PLoS One* (*n* = 38, IF2018 = 2.776, Q2), and *Archives of Toxicology* (*n* = 37, IF2018 = 5.741, Q1). The journals (21/592, 3.55%) with the publication number greater than or equal to 21 (T = 21) were used to construct the citation network map. **Figure 4A** shows that *Toxicological Sciences*, *Hepatology*, *Liver International*, and *PLoS One* have larger sizes of nodes because of their more publications. *Toxicological Sciences* has active citation relationships with *Archives of Toxicology*, *Chemical Research in Toxicology*, and *Journal of Applied Toxicology*, etc.; *Hepatology* has strong citation relationships with *Liver International*, *Journal of Hepatology* and *Seminars in Liver Disease*, etc. The two journals also have a co-citation relationship when they are cited simultaneously in one or more identical publications (Gao et al., 2019). Among 7,645 co-cited academic journals, five journals had ccitations over 2,000, and all of them were from the United States, except for the *Journal of Hepatology* from Netherlands (**Table 2**). *Hepatology* had the most co-citations (*n* = 7,383, IF2018 = 14.971, Q1), followed by *Gastroenterology* (*n* = 3,326, IF2018 = 19.233, Q1), *Journal of Hepatology* (*n* = 2,892, IF2018 = 18.946, Q1), *Toxicological Sciences* (*n* = 2,317, IF2018 = 3.564, Q1), and *Drug Metabolism and Disposition* (*n* = 2,062, IF2018 = 3.354, Q2) (**Table 2**). Among the top 15 co-cited journals, *the New England Journal of Medicine* has the highest IF (IF2018 = 70.67). The journals (21/7,645, 0.27%) with co-citations greater than or equal to 700 (T = 700) were used to construct the co-citation network. According to **Figure 4B**, *Hepatology* and *Gastroenterology* have larger node sizes owing to their more co-citations. *Hepatology* has active co-cited relationships with *Gastroenterology*, *Journal of Hepatology*, *Toxicological Sciences*, *Drug Metabolism and Disposition*, etc.

**Table 2:** The top 10 journals and co-cited journals of DILI research.

Rank	Journal	Count	IF2018^#^	Q^*^	Co-cited Journal	Co-citation	IF2018	Q
1	Toxicological Sciences (United States)	79	3.564	Q1	Hepatology (United States)	7383	14.971	Q1
2	Hepatology (United States)	48	14.971	Q1	Gastroenterology (United States)	3326	19.233	Q1
3	Liver International (United States)	38	5.542	Q1	Journal of Hepatology (Netherlands)	2892	18.946	Q1
4	PLoS One (United States)	38	2.776	Q2	Toxicological Sciences (United States)	2317	3.564	Q1
5	Archives of Toxicology (Germany)	37	5.741	Q1	Drug Metabolism and Disposition (United States)	2062	3.354	Q2
6	Clinics in Liver Disease (United States)	36	5.233	Q1	Chemical Research in Toxicology (United States)	1431	3.274	Q2
7	International Journal of Molecular Sciences (United States)	31	4.183	Q2	New England Journal of Medicine (United States)	1329	70.67	Q1
8	Chemical Research in Toxicology (United States)	30	3.274	Q2	Clinical Pharmacology & Therapeutics (United States)	1214	6.336	Q1
9	Toxicology Letters (Ireland)	30	3.499	Q2	Toxicology and Applied Pharmacology (United States)	1201	3.585	Q2
10	Journal of Hepatology (Netherlands)	29	18.946	Q1	American Journal of Gastroenterology (United States)	1138	10.241	Q1
11	Frontiers in Pharmacology (Switzerland)	28	3.845	Q1	Journal Pharmacology and Experimental Therapeutics (United States)	1131	3.615	Q1
12	Drug Safety (New Zealand)	27	3.526	Q2	Journal of Biological Chemistry (United States)	1060	4.106	Q2
13	World Journal of Gastroenterology (China)	27	3.411	Q2	PLoS One (United States)	1050	2.776	Q2
14	Expert Opinion on Drug Metabolism & Toxicology (England)	26	3.487	Q2	Proceedings of the national academy of Sciences of the United States of America (United States)	955	9.58	Q1
15	Toxicology in Vitro (England)	25	3.067	Q2	Drug Safety (New Zealand)	897	3.526	Q2

^#^IF: Impar Factor; *Q: Quartile in Category.

**Figure 5:**
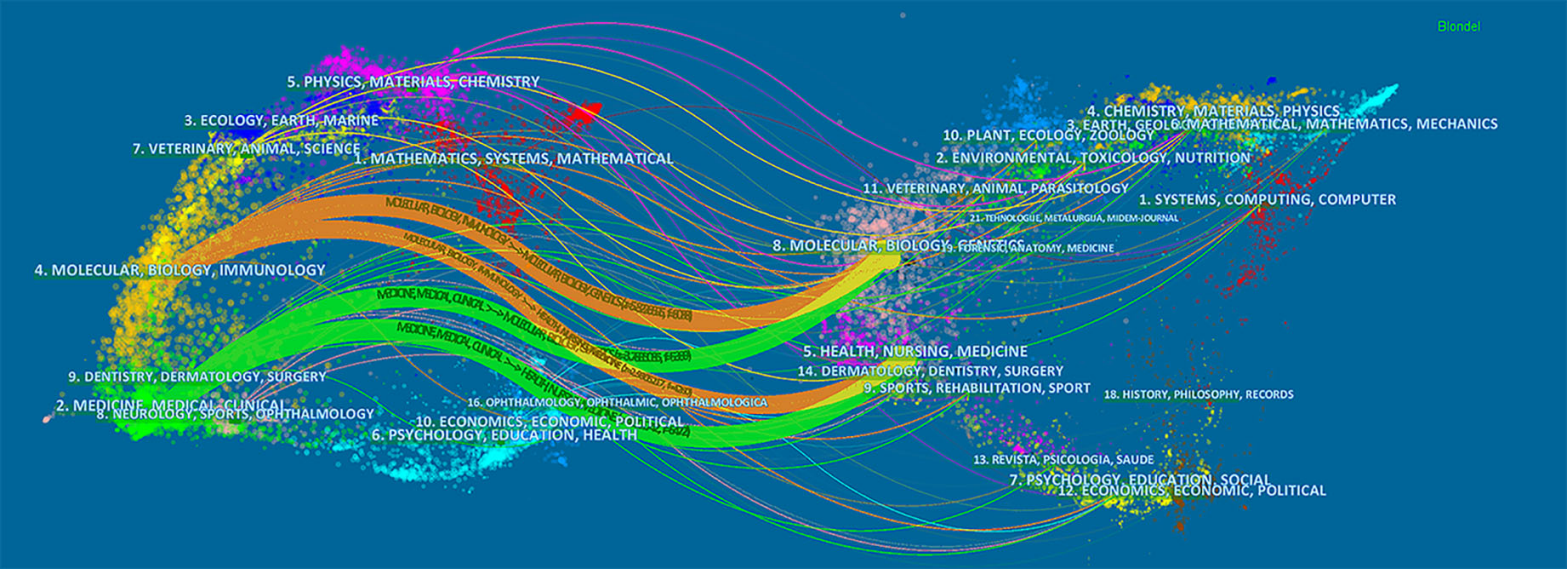
The dual-map overlay of journals related to drug-induced liver injury (DILI) research.

The dual-map overlay of journals stands for the topic distribution of the journals (**Figure 5**); the citing journals have been placed on the left side, cited journals on the right side, and the colored paths indicate the citation relationships (Chen, 2017; Miao et al., 2018). Four main citation paths were identified, two orange paths and two green paths. The orange paths indicate that the studies, published in Molecular/Biology/Genetics journals and Health/Nursing/Medicine journals, are usually cited in the studies, published in Molecular/Biology/Immunology journals. The green paths represent that studies, published in Molecular/Biology/Genetics journals and Health/Nursing/Medicine journals, are typically cited in the studies, published in Medicine/Medical/Clinical journals.

### Authors and Co-Cited Authors

**Figure 6:**
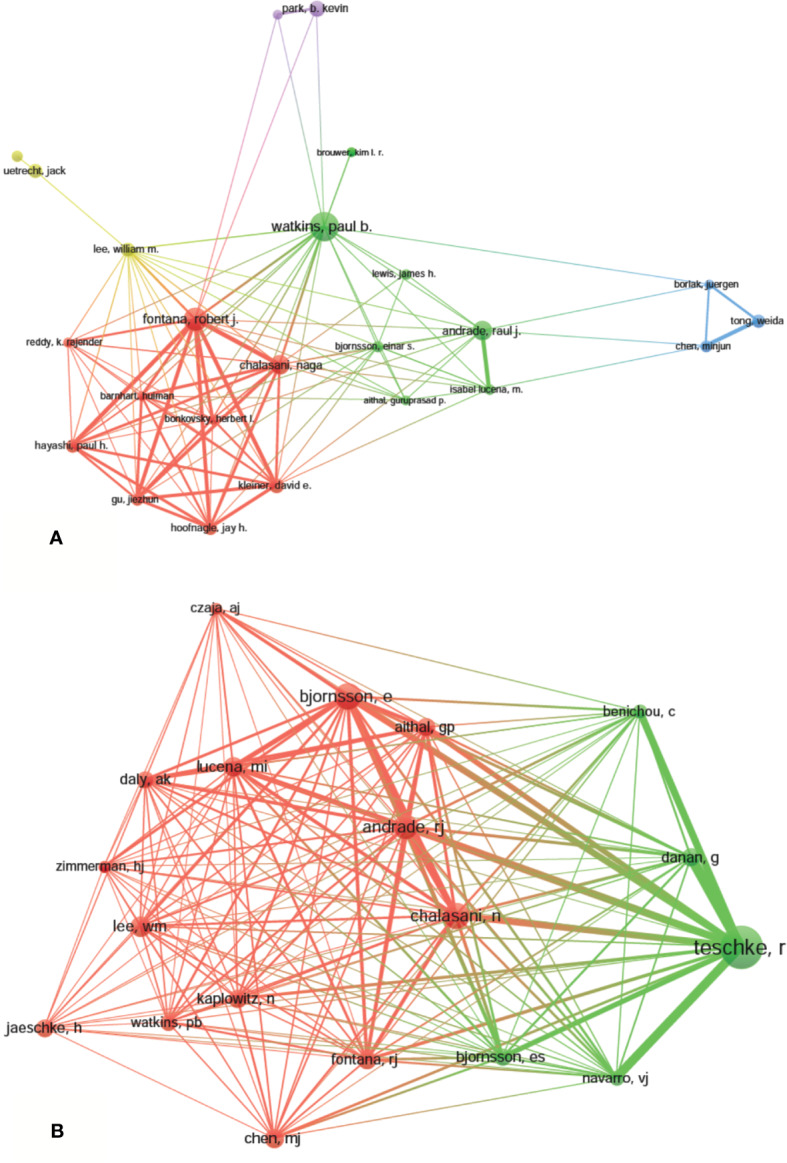
The network map of authors (**A**, T = 17) and co-cited authors (**B**, T = 230) related to drug-induced liver injury (DILI) research.

A total of 9,236 authors were involved in the DILI-related studies. Six authors published over 35 articles. Watkins PB published the most papers (*n* = 59) and ranked first, followed by Fontana RJ (*n* = 46) and Teschke R (*n* = 39). Yokoi T, Andrade RJ, and Chalasani N were ranked the fourth (*n*=37). The remaining four authors published 26 to 30 papers (**Table 3**). The authors (30/9,236, 0.32%) with the publication number greater than or equal to 17 (T = 17) were used to construct the co-authorship map. The largest subnetwork is presented in **Figure 6A**. The node sizes of Watkins PB and Fontana RJ are larger owing to their more publications (Teschke R is not in this subnetwork). Close cooperation was observed among several authors, such as Fontana RJ and Hayashi PH, Chalasani N and Hoofnagle JH, Andrade RJ and Isabel LM, etc.

**Table 3:** The top 10 authors and co-cited authors of DILI research.

Rank	Author	Count	Co-cited author	Co-citation
1	Watkins PB	59	Teschke R	945
2	Fontana RJ	46	Björnsson E	564
3	Teschke R	39	Chalasani N	548
4	Yokoi T	37	Andrade RJ	522
5	Andrade RJ	37	Lee WM	425
6	Chalasani N	37	Lucena MI	404
7	Park BK	30	Chen MJ	384
8	Kleiner DE	28	Aithal GP	383
9	Lee WM	26	Fontana RJ	382
10	Uetrecht J	26	Danan G	368

Co-cited authors are the authors, who have been co-cited in a range of publications (Li et al., 2017). Among 38,679 co-cited authors, four authors had co-citations over 500. Teschke R had the most co-citations (*n* = 945) and ranked the first, followed by Björnsson E (*n* = 564), Chalasani N (*n* = 548), and Andrade RJ (*n* = 522) (**Table 3**). The remaining six top authors had co-citations from 368 to 425. The authors (19/38,679, 0.05%) with co-citations greater than or equal to 230 (T = 230) were used to construct the co-citation picture. According to **Figure 6B**, Teschke R has the largest node size for the most co-citations and has active co-cited relationships with Danan G, Bénichou C, Navarro VJ, Björnsson E, Aithal G, Andrade RJ, and Chalasani N; Andrade RJ has strong co-cited relationships with Björnsson E, Chalasani N, Aithal G, Fontana RJ and Lucena MI, etc.

### Co-Cited References

**Figure 7:**
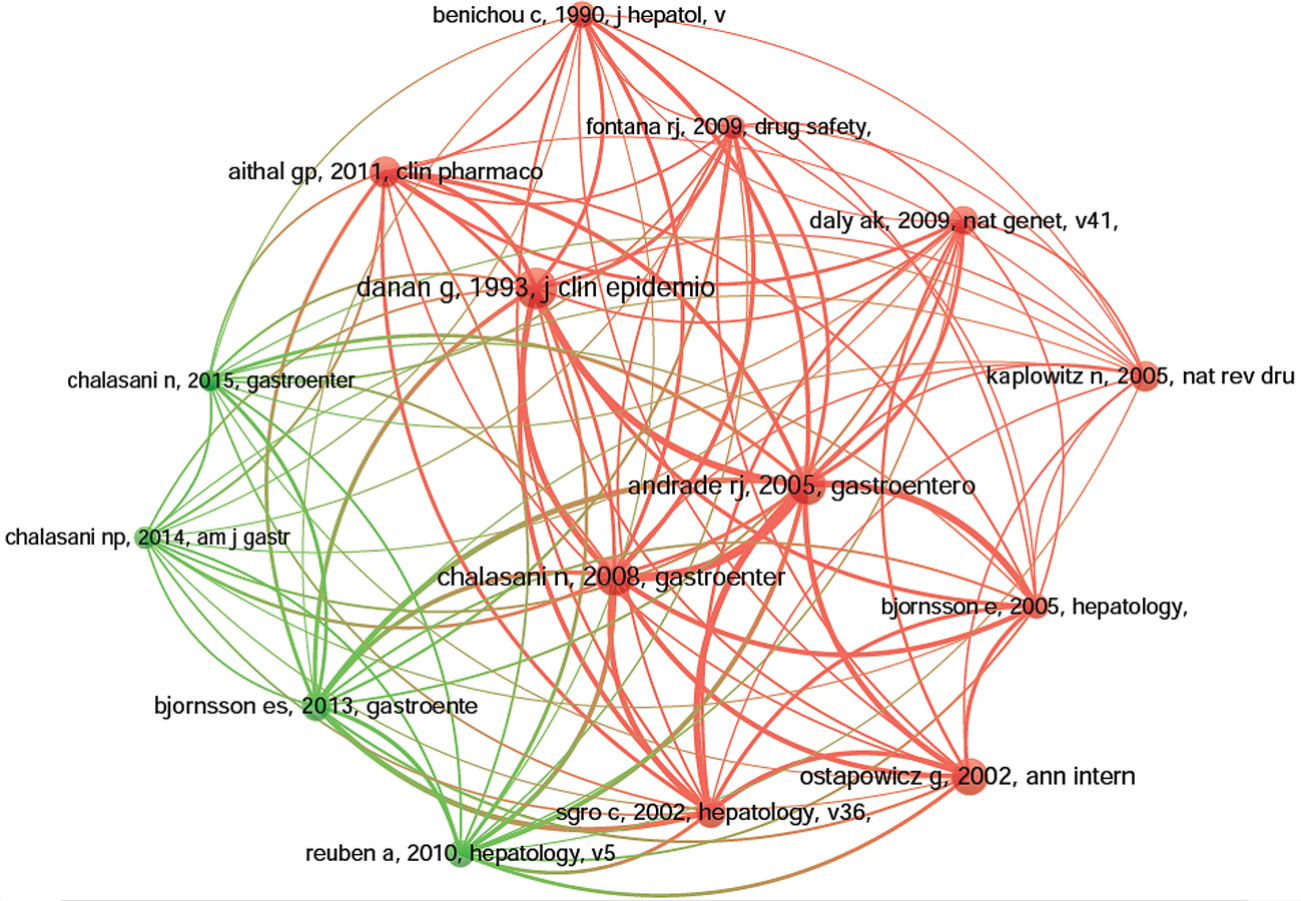
The network map of co-citation references for drug-induced liver injury (DILI) research (T = 125).

Co-cited references are those references, which are cited together by other publications (Lu et al., 2019b). Among 1995 DILI-related publications, there were 56,273 co-cited references. We have presented the top 10 co-cited references in **Table 4**. Each reference was co-cited at least 158 times, and four references were co-cited over 200 times. For example, Danan G et al. (Danan and Benichou, 1993) published a study, entitled “Causality assessment of adverse reactions to drugs-I. A novel method based on the conclusions of international consensus meetings: application to drug-induced liver injuries” in the *Journal of Clinical Epidemiology*, and this publication was co-cited the most number of times (*n* = 256) and ranked first, followed by Andrade RJ et al. (Andrade et al., 2005) in *Gastroenterology* (2005, *n* = 243), Chalasani N et al. (Chalasani et al., 2008) in *Gastroenterology* (2008, *n* = 237) and Ostapowicz G et al. (Ostapowicz et al., 2002) in *Annals of internal medicine* (2002, *n* = 222). The remaining six references had co-citations from 158 to 185 (**Table 4**). The references (15/56,273, 0.03%) with co-citations greater than or equal to 125 (T = 125) were used to construct the co-citation map. In **Figure 7**, “Danan G, 1993, *J Clin Epidemiol* (Danan and Benichou, 1993)“ with the largest size shows active co-cited relationships with “Andrade RJ, 2005, *Gastroenterology* (Andrade et al., 2005)“, “Chalasani N, 2008, *Gastroenterology* (Chalasani et al., 2008)“, “Björnsson ES, 2013, *Gastroenterology* (Björnsson et al., 2013)“, “Aithal GP, 2011, *Clin Pharmacol Ther* (Aithal et al., 2011)“, “Bénichou C, 1990, *J Hepatol* (Bénichou, 1990)“, etc. Several references, such as “Andrade RJ, 2005, *Gastroenterology* (Andrade et al., 2005)“, “Chalasani N, 2008, *Gastroenterology* (Chalasani et al., 2008)“, “Sgro C, 2002, *Hepatology* (Sgro et al., 2002)“, “Ostapowicz G, 2002, *Ann intern med* (Ostapowicz et al., 2002)“, “Björnsson E, 2005, *Hepatology* (Björnsson et al., 2013)“, and “Daly Ak, 2009, *Nat Genet* (Daly et al., 2009)“, were also simultaneously co-cited actively in other articles.

**Table 4:** The top 10 co-cited references related to DILI research.

Rank	Co-cited reference	Count
1	Danan G, 1993, J Clin Epidemiol, V46, P1323 (Danan and Benichou, 1993)	256
2	Andrade RJ, 2005, Gastroenterology, V129, P512 (Andrade et al., 2005)	243
3	Chalasani N, 2008, Gastroenterology, V135, P1924 (Chalasani et al., 2008)	237
4	Ostapowicz G, 2002, Ann intern med, V137, P947 (Ostapowicz et al., 2002)	222
5	Aithal GP, 2011, Clin Pharmacol Ther, V89, P806 (Aithal et al., 2011)	185
6	Kaplowitz N, 2005, Nat Rev Drug Discov, V4, P489 (Kaplowitz, 2005)	182
7	Sgro C, 2002, Hepatology, V36, P451 (Sgro et al., 2002)	181
8	Daly Ak, 2009, Nat Genet, V41, P816 (Daly et al., 2009)	173
9	Björnsson ES, 2013, Gastroenterology, V144, P1419 (Björnsson et al., 2013)	172
10	Bénichou C, 1990, J Hepatol, V11, P272 (Bénichou, 1990)	158

### References With Citation Burstness

**Figure 8:**
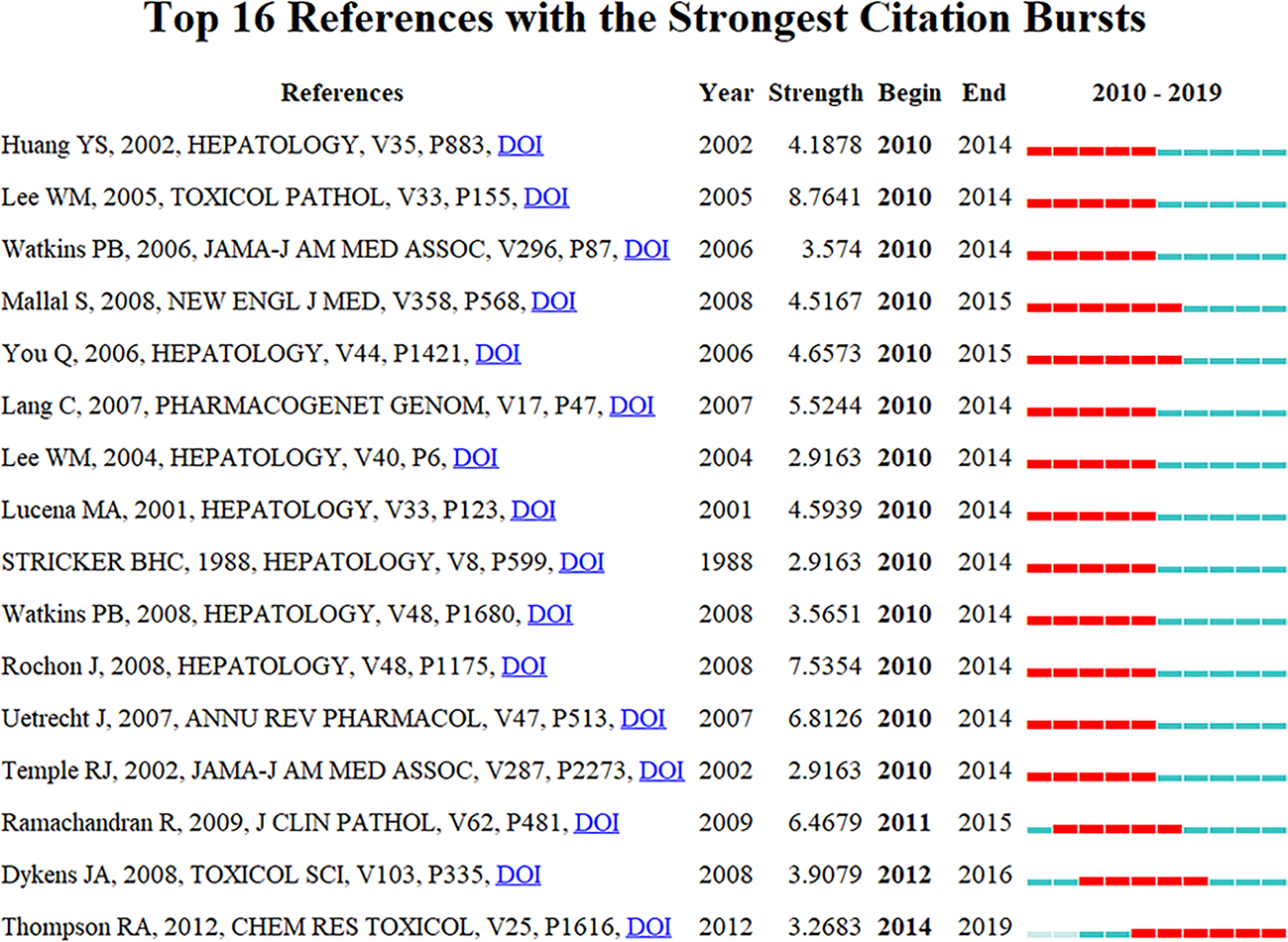
Top 16 references with strong citation burstiness (MD = 5). The red bars mean some references cited frequently; the blue bars were references cited infrequently.

Citation burstness are those references, which are often focused on closely by scholars in a specific field at an interval of time (Huang et al., 2019). In CiteSpace, the minimum duration of the burstness was set for five years for DILI-related publications, and 16 references were detected with strong citation burstness. In **Figure 8**, each red or blue bar represents the time interval, and a single bar is equal to one year. The red bar especially represents citation burstness (Huang et al., 2019). About 81.25% (13/16) of the references appeared citation burstness in 2010, and the strongest burstness (*n* = 8.7641) among the top 16 references was caused by the paper entitled “Recognizing Drug-Induced Liver Injury: Current Problems, Possible Solutions”, authored by Lee WM *et al*. (Lee and Senior, 2005) with citation burstness from 2010 to 2014. The remaining three references were detected with starting citation burstness after 2010 (2011, 2012, and 2014). The publication entitled “Histological patterns in drug-induced liver disease”, published in *Journal of Clinical Pathology* by Ramachandran R *et al*. (Ramachandran and Kakar, 2009), exhibited citation burstness from 2011 to 2015 (*n* = 6.4679), followed by “*In Vitro* Assessment of Mitochondrial Dysfunction and Cytotoxicity of Nefazodone, Trazodone, and Buspirone”, published in *Toxicological Sciences* by Dykens JA *et al*. (Dykens et al., 2008), which showed citation burstness from 2012 to 2016 (*n* = 3.9079), and “*In V*itro Approach to Assess the Potential for Risk of Idiosyncratic Adverse Reactions Caused by Candidate Drugs”, published in *Chemical Research Toxicology* by Thompson RA *et al*. (Thompson et al., 2012), displayed citation burstness from 2014 to 2019 (*n* = 3.2683). Overall, the burstness strength of the top 16 DILI references ranged from 2.9163 to 8.7641, while endurance strength was 5 or 6 years.

## Discussion

### General Information

Based on 2010–2019 data from SCIE in WoSCC, a total of 1,995 papers were in 592 peer-reviewed journals with 56,273 co-cited references in 10 languages by 2,331 institutions from 79 countries/regions, and the scientometric study included the analysis of the research status, development tendencies, and hotspots of the DILI-related publications to offer the references to researchers. The annual output of the DILI related publications was generally increasing in the past decade. According to the linear fitting, almost 340 studies will be published in 2020, suggesting that researches are now paying more attention to the DILI-related topics in recent years. The United States, China, and Japan were the top 3 productive countries and the United States was foremost because it contributed to 36.44% of all publications. Besides, the scientific research abilities of the developing countries in the field of DILI should be improved; because as a developing country, China is in the top 10 countries. Thus, developing countries should actively learn from developed countries and put forward ideas to advance research of DILI. About 60% of top institutions were from the United States. The University of North Carolina, the University of Michigan, and the University of Liverpool published the most, and we found active collaboration between the University of North Carolina and the University of Michigan, indicating their significant contribution to DILI-related research. The scholars could also develop cooperative relationships with those productive institutions. *Toxicological Sciences* published the majority of the studies, while *Hepatology* received the most co-citations. Meanwhile, the journals from the United States accounted for the largest proportion of the top 10 journals and co-cited journals (46.67% and 86.67%, respectively), indicating that journals in the United States contributed to many studies and arouse much attention. Moreover, we found more co-citations in high-IF journals, suggesting that these journals were more co-cited frequently and played important roles in the DILI-related researches. Top co-cited academic journals could be sources of references when we write manuscripts and productive academic journals could be taken into considerations when we submit manuscripts. The dual-map overlay of the journal analysis also can provide indispensable references for new researchers. Among the authors, Watkins PB published the majority of the studies, while Teschke R had the most co-citations. Additionally, we found that five authors, namely Fontana RJ, Teschke R, Andrade RJ, Chalasani N, and Lee WM, were not only the top 10 productive authors but also the top 10 co-cited authors, indicating that these five authors contributed significantly in this filed. Especially, Fontana RJ and Teschke R published more studies and received higher co-citations, suggesting that their teams could be better potential collaborators for researchers. These authors also had relatively stable cooperation. Among the top 10 productive authors and top 10 co-cited authors, Andrade RJ, Chalasani N, Aithal GP, and Danan G also participated in four of the co-cited references.

### Knowledge Base

Co-cited references represent how frequently two publications are cited together by other publications, therefore they can be viewed as a knowledge base in a particular field (Chen, 2006). In this scientometric review, the top 10 co-cited references were selected to identify the knowledge base of DILI. Danan G et al. published the most co-cited study in 1993, with 256 co-citations (Danan and Benichou, 1993). This study provided a novel causality assessment of drug-related adverse reactions (especially, DILI). The second co-cited publication was published in *Gastroenterology* by Andrade RJ et al. (Andrade et al., 2005). They performed an analysis of 461 cases over 10 years and found that 11.7% of the patients with drug-induced hepatocellular jaundice might die or undergo transplantation; amoxicillin-clavulanate contributed to the majority of the DILI-related cases. In 2008, Chalasani N et al. (Chalasani et al., 2008) published the third co-cited paper in *Gastroenterology*. This prospective study summarized the causes, clinical features, and outcomes from a total of 300 patients, and it reported that DILI was caused by various drugs, herbs, and dietary supplements; antibiotics were the major contributor to DILI. Moreover, HCV RNA testing should be used to exclude acute HCV infection in patients with suspected DILI. *Annals of Internal Medicine* published the fourth co-cited prospective study by Ostapowicz *et al*. (Ostapowicz et al., 2002) in 2002. This study showed that excessive use of acetaminophen and idiosyncratic drug reactions had replaced viral hepatitis as the most common cause of acute hepatic failure at 17 tertiary care centers in the United States. The fifth co-cited publication was published in 2011 by Aithal GP et al. (Aithal et al., 2011); it provided the case definition and phenotype standardization of DILI. The sixth co-cited study was published in 2005 by Kaplowitz N et al. (Kaplowitz, 2005). This review summarized the clinical signatures and pathophysiology (focusing on the characteristics of the innate immune system and the regulation of cell-death pathways) of idiosyncratic drug hepatotoxicity. Moreover, they discussed the predictive signals of DILI and procedures to prevent the failure of preclinical toxicology by predicting idiosyncratic reactions. The seventh most commonly co-cited paper was published by Sgro C et al. (Sgro et al., 2002) in *Hepatology* in 2002. This French population-based study concluded that the morbidity and severity of drug-induced hepatitis in the general population were significantly underrated, and the key drugs were nonsteroidal antiinflammatory drugs (NSAIDs), psychotropic, antiinfectious and hypolipidemic agents. In 2009, the eighth most commonly co-cited paper was published by Daly AK *et al*. (Daly et al., 2009) in *Nature Genetics*. They conducted a genome-wide association (GWA) study and found that the HLA-B*5701 genotype was a key determinant of flucloxacillin-induced DILI. Björnsson ES et al. (Björnsson et al., 2013) analyzed the morbidity and the quantitative risk of DILI in Iceland and published the ninth co-cited paper. They reported that amoxicillin-clavulanate was the most common contributor to DILI, and both azathioprine and infliximab were potentially hepatotoxic. The consensus, published in *Journal of Hepatology* in 1990, received the last co-citations; it proposed standard designations of DILI and criteria of causality assessment, which reflected in its titles “Criteria of Drug-Induced Liver Disorders: Report of an International Consensus Meeting” (Bénichou, 1990). Generally, the top 10 co-citations were focused on the following subjects: definition, incidence rate or clinical characteristics, etiology or pathogenesis (such as the character of the innate immune system, the regulation of cell-death pathways and susceptible HLA-B*5701 genotype), identification of the main drugs, and causality assessment (criteria and methods) of DILI, all which were the foundations of DILI research.

### Emerging Topics

The publications with strong citation burstness are followed closely by the researchers over a time-period (Chen, 2017; Miao et al., 2018). Thus, the emerging topics in DILI-related researches were represented by them. The top 16 references with strong strength citation burstness were identified by CiteSpace. The citation burstness of 11 (Stricker et al., 1988; Lucena et al., 2001; Huang et al., 2002; Temple and Himmel, 2002; Lee, 2004; Lee and Senior, 2005; Watkins et al., 2006; Lang et al., 2007; Uetrecht, 2007; Rochon et al., 2008; Watkins et al., 2008) of the top 16 references lasted from 2010 to 2014, two references (You et al., 2006; Mallal et al., 2008) from 2010 to 2015, one reference (Ramachandran and Kakar, 2009) from 2011 to 2015, one reference (Dykens et al., 2008) from 2012 to 2016, and one reference (Thompson et al., 2012) from 2014 to 2019. Among eleven references with citation burstness lasting from 2010 to 2014, the top 3 references with the strongest citation burstness were published by Lee WM et al. (Lee and Senior, 2005) (2005, *Toxicologic Pathology*, *n* = 8.7641), Rochon J et al. (Rochon et al., 2008) (2008, *Hepatology*, *n* = 7.5354), and Uetrecht J *et al*. (Uetrecht, 2007) (2007, *Annual Review of Pharmacology and Toxicology*, *n* = 6.8126). Lee WM et al. (Lee and Senior, 2005) mainly discussed two issues: 1) whether the drug or another process caused hepatic diseases and 2) the clinical risk factors and the true incidence of drug-induced hepatotoxicity. Rochon J et al. (Rochon et al., 2008) explored the dependability of the “Roussel Uclaf Causality Assessment Method” (RUCAM) for evaluating the causality of DILI. They found that the middling reliability of the RUCAM was challenging for future studies on DILI, and different approaches, including adapting the RUCAM, advancing drug-specific apparatuses, or assessing the causality development on expert opinions, could be more suitable. Uetrecht J et al. (Uetrecht, 2007) discussed the idiosyncratic drug reactions (IDRs), including definition, clinical characteristics, genetic associations, mechanistic hypotheses, animal models, and the roles of mRNA profiles, proteomics, and metabolomics in studies of IDRs. The remaining eight publications (Stricker et al., 1988; Lucena et al., 2001; Huang et al., 2002; Temple and Himmel, 2002; Lee, 2004; Watkins et al., 2006; Lang et al., 2007; Watkins et al., 2008) similarly discussed the above-mentioned three topics with small differences, such as screening and diagnosis of susceptible genotyping by Huang YS et al. (Huang et al., 2002) in *Hepatology* and Lang C et al. (Lang et al., 2007) in *Pharmacogenetics and Genomics*, prevention of the drugs causing DILI by Watkins PB et al. in *JAMA* (Watkins et al., 2006) and *Hepatology* (Watkins et al., 2008), Lee WM et al. (Lee, 2004) in *Hepatology*, Stricker BHC et al. (Stricker et al., 1988) in *Hepatology* and Temple RJ et al. (Temple and Himmel, 2002) in *JAMA*, causality assessment in the DILI-related cases by Lucena MA et al. (Lucena et al., 2001) in *Hepatology*.

The citation burstness of five publications (You et al., 2006; Dykens et al., 2008; Mallal et al., 2008; Ramachandran and Kakar, 2009; Thompson et al., 2012) ended in 2015 or later. They represented the latest emerging themes of DILI and were considered for further discussions. The first paper, ranked by burstness strength (*n* = 6.4679), was published by Ramachandran R et al. (Ramachandran and Kakar, 2009) in 2009, and the citation burstness lasted for 4 years (2011–2015), summarizing histological patterns in DILI. They concluded that “acute hepatitis, with or without cholestasis, was the most common histological pattern of DILI, and drugs, such as acetaminophen, were the foremost reasons for acute liver failure”. You Q et al. (You et al., 2006) developed a mouse model for halothane-induced liver injury and demonstrated the vital roles of neutrophils in the progress of DILI. The study was published in *Hepatology* in 2006 with the second strongest citation burstness (*n* = 4.6573) lasting for six years (2010–2015). The publication with the third strongest citation burstness was published in *the New England and Journal of Medicine* by Mallal S et al. (Mallal et al., 2008) in 2008 with the burstness strength of 4.5167, and the burstness lasted for 6 years (2010–2015). Genetic susceptibility by human leucocyte antigen (HLA) alleles is the risk factor for DILI. A double-blind, prospective, randomized study was conducted to prevent hypersensitivity (hepatitis, fever, or rash) to abacavir by prospective HLA-B*5701 screening; this study highlighted the role of genetic pharmacology tests in preventing adverse drug reactions. This paper exhibited the fourth-highest citation burstness by Dykens JA et al. (Dykens et al., 2008) in 2008, and the authors assessed the cytotoxicity and mitochondrial dysfunction of nefazodone, trazodone, and buspirone, which probably led to hepatotoxicity. This paper had a burstness strength of 3.9079. Finally, Thompson RA et al. (Thompson et al., 2012) published the article with the fifth-highest citation burstness (*n* = 3.2683) in *Chemical Research in Toxicology* in 2012, and it lasted from 2014 until 2019. In this study, the possible risks of idiosyncratic adverse reactions (IADRs) induced by drug candidates were evaluated by an *in vitro* method, and they proposed that this integrated method could select the potential candidate drugs with a decreased tendency to cause IADRs in humans. The citation burstness analysis showed that exploring the microscopic mechanism of DILI (such as organelle dysfunction and cytotoxicity causing by drugs, exploration of the role of neutrophils in DILI using mouse models) and developing newer approaches to prevent DILI (such as the prospective HLA-B*5701 screening, *in vitro* approaches for assessing the potential risk of candidate drugs for DILI) were the recent major topics in the field of DILI research.

### Strengths and Limitations

Our study has several unique strengths. Firstly, we used the scientometric method to systematically analyze the DILI-related publications for the first time, which could comprehensively guide the clinicians and scholars focusing on DILI. Secondly, we used four scientometric tools to performed this investigation simultaneously, and three of them (HistCite, VOSviewer, and CiteSpace) have been widely used in the scientometric field (Gao et al., 2019; Huang et al., 2019; Lu et al., 2020). Thus, the data analysis process may be objective. Thirdly, compared to traditional narrative reviews, the scientometric analysis provides a better insight into the evolving research foci and trends (Huang et al., 2019). Like other scientometric researches, our study has several limitations. Firstly, data were retrieved from only WoSCC, instead of searching other databases like Embase or Scopus. But we need to note that WoSCC is the most commonly applied tool for scientometric analyses (Miao et al., 2018; Gao et al., 2019). Moreover, current scientometric tools face extreme difficulties in analyzing data from multiple databases simultaneously. Secondly, all information was extracted by scientometric tools rather than authors manually in meta-analysis or overview of systematic review (Nugent et al., 2017; Pan et al., 2018). Thus, the bias of our results may also exist. For example, the possibility of homonyms of authors would not be excluded, but these data cannot be obtained accurately by these existing tools. These problems may be solved in the future with the development of machine learning, natural language processing, and data science (Kehl et al., 2019). Lastly, the publications in 2020 were not included because of the inadequate data.

## Conclusion

We used HistCite, CiteSpace, VOSviewer, and R-bibliometrix to analyze the knowledge base and research hotspots on DILI publications in the past decade. The United States contributed the most in the DILI-related researches, and the University of North Carolina produced the most publications. *Toxicological Sciences* and *Hepatology* were the significant journals for DILI. Fontana RJ and Teschke R may have an important influence on DILI research, because they published numerous articles on DILI and were co-cited in several more publications. Definition, incidence rate or clinical characteristics, etiology or pathogenesis, identification of the main drugs, causality assessment of DILI were the knowledge base of DILI-related research. Exploring the microscopic mechanism and developing new approaches to prevent DILI were the recent topics in DILI research. This scientometric review offers a comprehensive understanding of DILI-related publications from 2010 to 2019, which could supply the references to the researchers in this field. As investigators discover more information on DILI, we expect that the prevention, diagnosis, management, and prognosis of DILI will soon become more effective and efficient.

## Data Availability Statement

The raw data supporting the conclusions of this article will be made available by the authors, without undue reservation.

## Author Contributions

YH, CL, and LK designed this study. LK and RS performed the search. LK and TL collected data. BM and CL rechecked data. CL and LK performed analysis. All authors wrote and reviewed this manuscript.

## Funding

This study was supported by grants from the Science and Technology Project of Guangzhou city (no. 201707010387).

## Conflict of Interest

The authors declare that the research was conducted in the absence of any commercial or financial relationships that could be construed as a potential conflict of interest.
