# Supplementary material for: Knowledge Mapping of Drug-Induced Liver Injury: A Scientometric Investigation (2010–2019)
Source: Front Pharmacol. 2020 Jun 5;11:842. doi: 10.3389/fphar.2020.00842 (PMC7291871; doi:10.3389/fphar.2020.00842)

**Appendix**

**Search strategy in Web of Science Core Collection (March 15, 2020)**

Topic = "drug induced liver injury" OR "drug induced liver damage" OR "drug induced liver disease" OR "drug induced liver diseases" OR "drug induced liver hepatitis" OR "drug induced liver hepatitides" OR "drug induced liver cirrhosis" OR "drug induced liver failure" OR "drug-induced liver injury" OR "drug-induced liver damage" OR "drug-induced liver disease" OR "drug-induced liver diseases" OR "drug-induced liver hepatitis" OR "drug-induced liver hepatitides" OR "drug-induced liver cirrhosis" OR "drug-induced liver failure" OR "herbal induced liver injury" OR "herbal induced liver damage" OR "herbal induced liver disease" OR "herbal induced liver diseases" OR "herbal induced liver hepatitis" OR "herbal induced liver hepatitides" OR "herbal induced liver cirrhosis " OR "herbal induced liver failure" OR "herbal-induced liver injury" OR "herbal-induced liver damage" OR "herbal-induced liver disease" OR "herbal-induced liver diseases" OR "herbal-induced liver hepatitis" OR "herbal-induced liver hepatitides" OR "herbal-induced liver cirrhosis " OR "herbal-induced liver failure" OR "herb induced liver injury" OR "herb induced liver damage" OR "herb induced liver disease" OR "herb induced liver diseases" OR "herb induced liver hepatitis" OR "herb induced liver hepatitides" OR "herb induced liver cirrhosis" OR "herb induced liver failure" OR "herb-induced liver injury" OR "herb-induced liver damage" OR "herb-induced liver disease" OR "herb-induced liver diseases" OR "herb-induced liver hepatitis" OR "herb-induced liver hepatitides" OR "herb induced liver cirrhosis" OR "herb-induced liver failure".


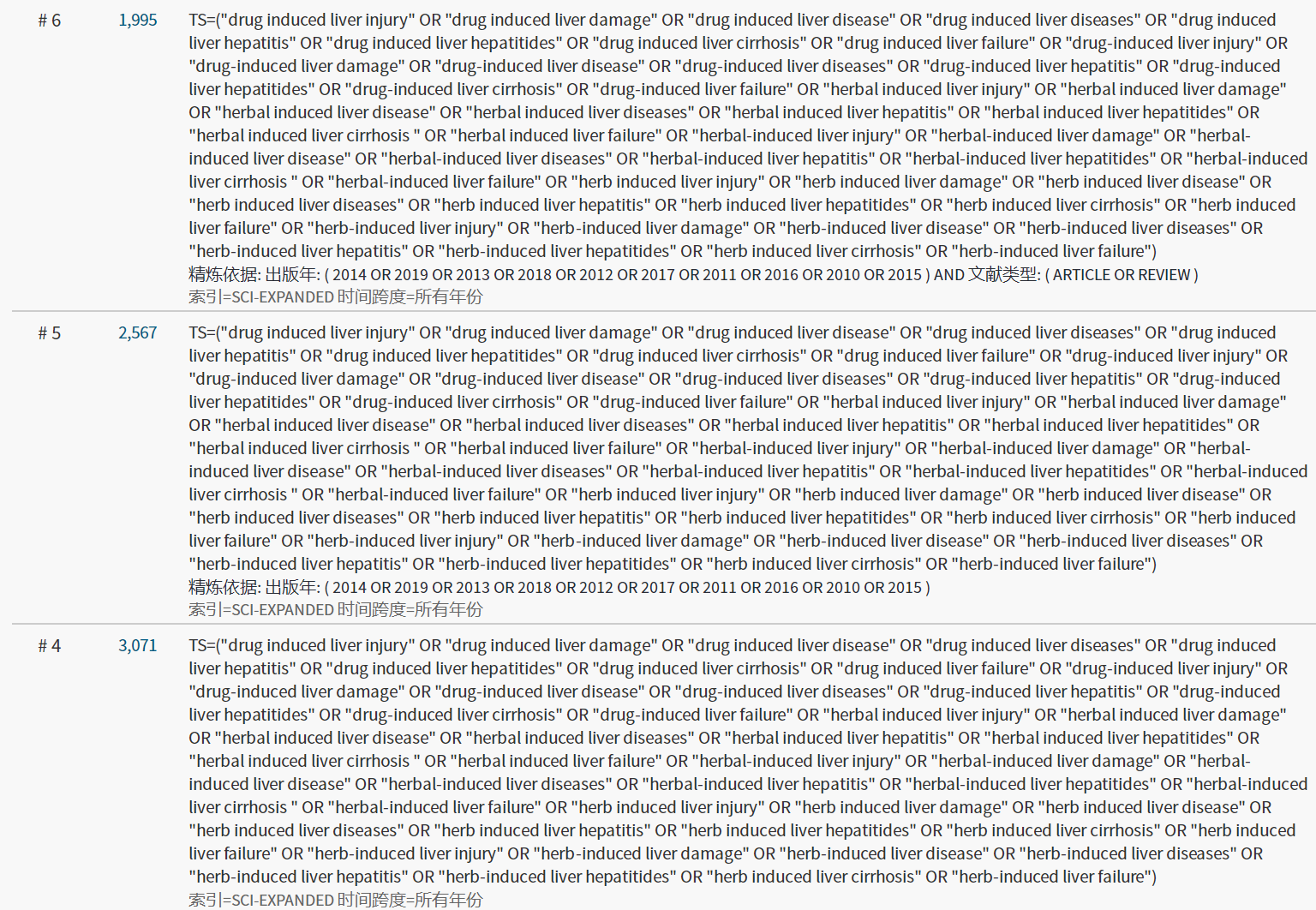

Supplement: Supplementary file 1 [file DataSheet_1.docx]
